# Cell Shape Dynamics: From Waves to Migration

**DOI:** 10.1371/journal.pcbi.1002392

**Published:** 2012-03-15

**Authors:** Meghan K. Driscoll, Colin McCann, Rael Kopace, Tess Homan, John T. Fourkas, Carole Parent, Wolfgang Losert

**Affiliations:** 1 Department of Physics, University of Maryland, College Park, Maryland, United States of America; 2 Laboratory of Cellular and Molecular Biology, National Cancer Institute, National Institutes of Health, Bethesda, Maryland, United States of America; 3 Department of Chemistry and Biochemistry, University of Maryland, College Park, Maryland, United States of America; 4 Institute for Physical Science and Technology, University of Maryland, College Park, Maryland, United States of America; North Carolina State University, United States of America

## Abstract

We observe and quantify wave-like characteristics of amoeboid migration. Using the amoeba *Dictyostelium discoideum*, a model system for the study of chemotaxis, we demonstrate that cell shape changes in a wave-like manner. Cells have regions of high boundary curvature that propagate from the leading edge toward the back, usually along alternating sides of the cell. Curvature waves are easily seen in cells that do not adhere to a surface, such as cells that are electrostatically repelled from surfaces or cells that extend over the edge of micro-fabricated cliffs. Without surface contact, curvature waves travel from the leading edge to the back of a cell at ∼35 µm/min. Non-adherent myosin II null cells do not exhibit these curvature waves. At the leading edge of adherent cells, curvature waves are associated with protrusive activity. Like regions of high curvature, protrusive activity travels along the boundary in a wave-like manner. Upon contact with a surface, the protrusions stop moving relative to the surface, and the boundary shape thus reflects the history of protrusive motion. The wave-like character of protrusions provides a plausible mechanism for the zig-zagging of pseudopods and for the ability of cells both to swim in viscous fluids and to navigate complex three dimensional topography.

## Author Summary

Migration of cells on surfaces and through tissues is an important part of life, from the amazingly coordinated migration of cells during development to the uncontrollable migration of metastatic cancer cells. Here we investigate the physics of cell migration with the goal of gaining new insights into how cells move and how they respond to obstacles. Through detailed quantitative analysis of time-dependent cell shapes, we demonstrate the existence of wave-like dynamic shape changes during the migration of *Dictyostelium discoideum*. Specifically, we observe that in migrating cells local protrusions propagate from the front toward the back along roughly alternating sides of the cell. Near the leading edge, protrusions that have not yet adhered to the surface move faster than the cell migration speed. We also investigated cells that were not in contact with a surface and cells extended over cliff edges. Such cells exhibit similar protrusion dynamics at their leading edges but, since in this case the protrusions cannot adhere to a surface, the associated boundary curvature waves continue to travel along the sides of the cells. While our study shows wave-like dynamics under many different conditions, we do not directly measure whether waves involve transport of membrane or intracellular material.

## Introduction

During chemotaxis, a chemical gradient directs cell migration. Chemotaxis occurs in phenomena as diverse as wound healing [1] and embryonic development [2], and has also been implicated in a wide array of pathological processes including aberrant angiogenesis [3] and cancer metastasis [4]. Chemotaxing cells can migrate individually during immune responses and neuronal patterning, or in cooperative groups during embryogenesis, wound healing, and organ and vasculature formation.

The social amoeba *Dictyostelium discoideum* is a model system for directed cell migration, and has been used to elucidate the regulatory processes of chemotaxis. *Dictyostelium* chemotaxis is of comparable speed to neutrophil chemotaxis and involves similar regulatory processes [5]. The chemotaxis of *Dictyostelium* is also more than an order of magnitude faster than typical epithelial cell migration and does not involve mature focal adhesion complexes [6]. *Dictyostelium* cells migrate directionally in gradients of cyclic adenosine monophosphate (cAMP). The binding of cAMP to specific cell surface receptors leads to the activation of various effectors, including adenylyl cyclase, resulting in the production and secretion of additional cAMP and the relay of the signal to neighboring cells [7]. This signal relay guides the cells to migrate collectively in a head-to-tail fashion toward aggregation centers and to maintain a preferred migration direction over distances much larger than the characteristic diffusion lengths of molecules [8]. Cells with the *aca* ^−^ mutation do not produce cAMP and, in its absence, are mostly round and immotile [9]. However, when stimulated with exogenous cAMP, *aca* ^−^ cells polarize and begin to migrate. If stimulated with a uniform concentration of cAMP, *aca* ^−^ cells exhibit chemokinesis (chemically induced random migration). In response to a cAMP gradient, these cells chemotax effectively [9].

The regulatory processes behind *Dictyostelium* chemotaxis have been investigated in great depth. Recent discoveries demonstrate the complexity of key chemotactic signaling pathways, including multiple compensatory mechanisms for sensing the direction of cAMP gradients [10]. Models of cell movement often consider migration as the final step in this process and use the output of the directional sensing machinery to assign a probability of forming a protrusion (and subsequently migrating) in a particular direction [11]. In most models, the location at which a new protrusion forms determines the direction of the protrusion and ultimately the direction of migration of the cell. For example, Haastert and Bosgraaf found that pseudopods grow in a direction perpendicular to the local boundary [12]. Directional migration in response to chemotactic signals is considered to be due to either the formation of new protrusions (the compass model) or the biased bifurcation of existing protrusions (the bifurcation and bias model) [13].

Quantitative studies of cell shape and motion that follow protrusions are now emerging, and indicate that protrusion dynamics are richer than anticipated [12], [14], [15], [16], [17], [18]. Machacek and Danuser, tracking a fixed region of the boundary of slow epithelial cells with sub-pixel resolution, found three distinct states of local protrusion activity: local protrusions that grow and retract in a single location along the cell edge, waves that travel along the edge of the cell, and fast, large-scale protrusions [14]. Dobereiner *et al.* found that wave-like protrusion dynamics are common to a wide range of slow-moving cells [19], especially during cell spreading.

How are such complex protrusion dynamics relevant to chemotaxis and directed cell migration? For the models of chemotaxis reviewed above, and for fast migration, a local protrusion phenotype is usually assumed in which protrusions are a (noisy) output in response to a chemotactic signal. This view appears to be supported by experiments in which a strong chemotactic signal is placed in close proximity to individual cells, causing a pseudopod to form in the direction of the signal [7].

However, recent experiments and modeling efforts point to a more complex protrusive machinery. Indeed, given signal strengths at physiological levels (and even in the absence of chemotactic gradients), protrusions tend to form on alternating sides of the leading edge of the cell, resulting in a zig-zag migration pattern [17]. Such zig-zag motion indicates that protrusion locations are not just described by a noisy output based on a chemical signal, but are also influenced by the prior protrusion history. The alternating position of pseudopods can be explained, for example, by considering the protrusive machinery as an excitable system [20].

The conclusion that alternating pseudopods are prominent in fast migrating cells relies on thresholds to separate individual pseudopods in a consistent way. However, it is unclear whether the underlying biology of protrusions justifies such thresholding. Instead, zig-zagging and alternating of pseudopods may be the result of wave-like behavior of the protrusive machinery. In developing tissues, actin waves can be seen to propagate across groups of cells [21]. There is also direct evidence that wave-like intracellular actin polymerization processes are prominent in fast migrating cells. Some of the first indications of this effect were from Vicker and colleagues, who found that *Dictyostelium* pseudopod dynamics are not random [22], but rather are associated with actin filament polymerization waves [23] that can drive locomotion [24]. Others were able to observe wave-like dynamics directly in actin polymerization and depolymerization [25]. The connection between internal waves and forces has also been elucidated [26]. Finally, the interaction between waves and surfaces is key to understanding migration: For example, Weiner found that when a neutrophil runs into another cell or an obstacle, actin waves are extinguished at the interaction site, allowing cells to change direction and avoid the obstacle [27]. This observation indicates that waves may not be observed when cells are confined between boundaries, such as e.g. in a key paper that reported zig-zagging of *Dictyostelium* protrusions [28].

To study the character of protrusions during fast cell migration and chemotaxis, we present new methods for the quantification of the dynamic shape of migrating cells. Using these methods, we demonstrate that protrusions in *Dictyostelium* have a wave-like character. As found in neutrophils, the waves appear to stop moving when touching the surface, so the wave-like character of protrusions is most evident when cells do not touch the surface, or extend over the edge of a cliff. While our study highlights that wave-like dynamics exist under many different conditions, we do not directly measure motion of the actin cytoskeleton or membrane, and thus have no conclusive evidence whether the wave-like dynamics reflect reaction-diffusion waves (due to actin polymerization or myosin contraction dynamics) or transport of membrane or intracellular material. These results could lead to a model that relates protrusive waves to the zig-zag-like appearance of cell tracks and the directional persistence of cell motion during chemotaxis.

## Results

### Regions of high boundary curvature move from the front to the back of cells

**Figure 1:**
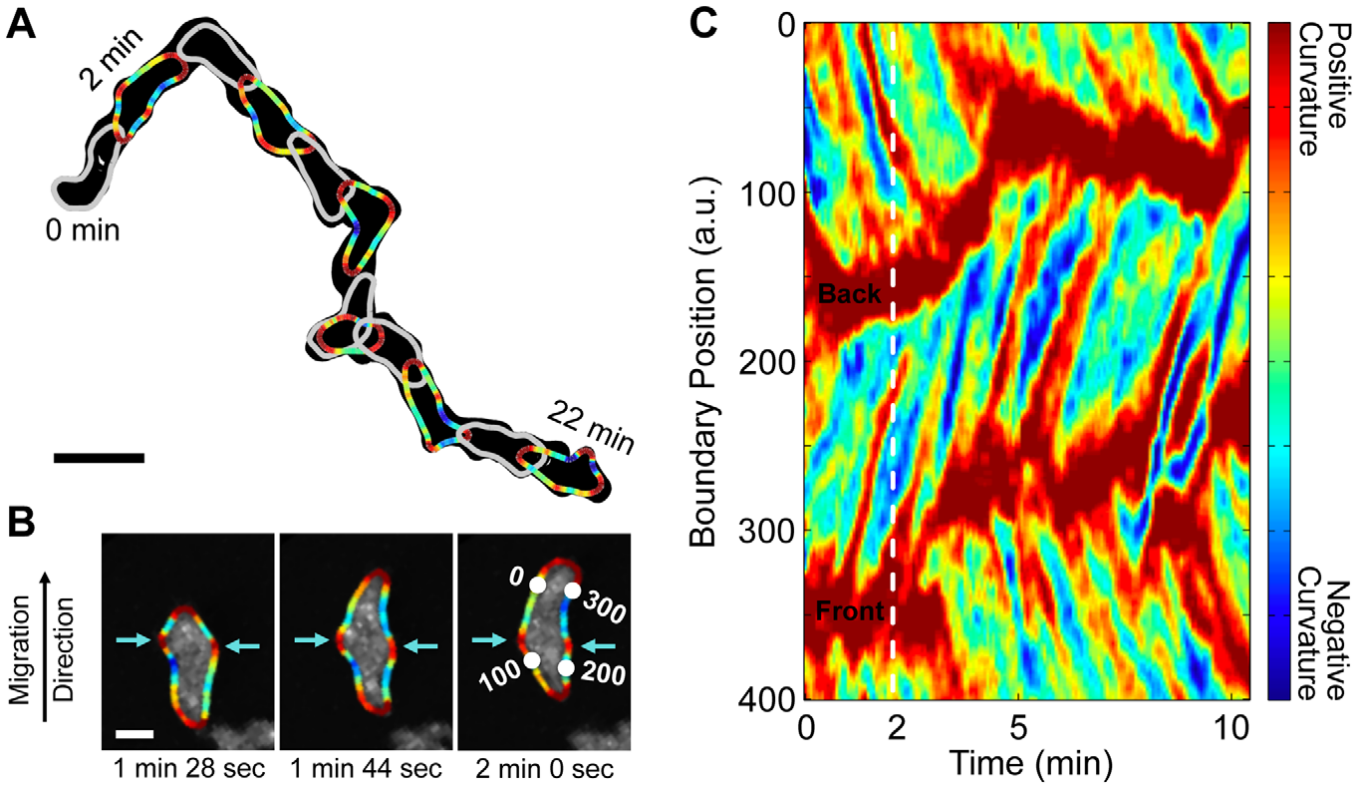
The boundary curvature of a self-aggregating wild-type *Dictyostelium* cell. (A) The overlapped light gray boundaries show the shape every 4 seconds, while the alternating dark gray and colored boundaries show the shape every 2 minutes. Colored boundaries represent curvature. (Scale bar, 20 µm.) (B) The boundary curvature overlaid on the original fluorescence images (*video S1*). Two curvature peaks, indicated by teal arrows, remain at the same location as the cell migrates through them. Numbers label the indices of every 100^th^ boundary point (*video S2*). (Scale bar, 5 µm.) (C) The spatial and temporal evolution of boundary curvature can be visualized in a kymograph. Peaks in boundary curvature propagate from the cell front (initially near boundary point 150) to the cell back (initially near boundary point 350).

To study the changing shape of migrating *Dictyostelium* cells, we used a snake (also known as active contour) algorithm. We analyzed the shapes of starved, wild-type (WT) cells that were self-aggregating. The extracted shape of one such migrating cell is shown in *figure 1a*. The boundary color represents curvature, a measure of local shape. As expected, the front and back of the cell have high curvature. Additional peaks in boundary curvature indicate other local protrusions. We find that additional protrusions first become visible near the front of the cell and, with respect to the cell, propagate towards the back as the cell migrates. At the sides of cells, these curvature bumps appear stationary relative to the surface (see *figure 1b* for a representative image sequence).

To study the motion and evolution of these propagating curvature peaks, we labeled the boundary in order to follow individual boundary points as they move. We defined a 1∶1 mapping between the boundary points in successive frames, choosing the mapping that minimized the total boundary point displacement. To create such a 1∶1 mapping, we described the boundary with the same number of points in every frame (400), even though the length of the perimeter of the cell varies from frame to frame.

With the boundary points labeled in this way, we are able to visualize and analyze how local properties of the boundary, such as curvature, vary as a function of both space and time. *Figure 1c* is a kymograph that depicts how curvature evolves along the boundary as a function of time. The front and back of the cell each appear as thick stripes of high curvature and the additional, lateral peaks appear as thin lines that connect the front to the back. Since the thin lines representing the curvature peaks are approximately parallel, the curvature peaks travel at equal speed with respect to the cell. The boundary curvature kymograph of an additional, developed, WT cell is shown in *figure S1a* (*video S3*). Averaging across 26 cells, we find that the peaks move relative to the cell at 10.9±0.7 µm/min, comparable to the average migration speed of *Dictyostelium* cells. This finding is consistent with the observation that peaks of high curvature on the side of the cell are stationary with respect to the surface.

We have previously found that alternative tracking mappings yield similar results, but generally do not track the entire local boundary [29]. For instance, kymographs can be constructed with boundary points that are a constant distance from neighboring boundary points, by using the boundary point that is closest to 0° relative to the center of the cell to align the boundaries from frame to frame. Such a mapping shows similar curvature peak patterns, but these kymographs are more difficult to interpret since only part of the local boundary is tracked from frame to frame [29].

*Figure 1c* shows that most high curvature regions on the side of cells start at the leading edge, and thus that the shape of the cell reflects a history of activity at the leading edge. Indeed we find a connection between curvature and protrusions at the leading edge, as described below. The amplitude of each peak varies as it travels from front to back, but tends to diminish with time, a further indication that the associated local protrusions are passive or shrinking. The local protrusions appear on roughly alternating sides, resulting in a loose right-left-right-left pattern that may reflect the zig-zag pattern of pseudopod activity. The pattern is not without defects, as multiple protrusions can travel simultaneously along the same side of the cell.

### Cell-surface contact is enhanced near curvature peaks at the sides of cells

**Figure 2:**
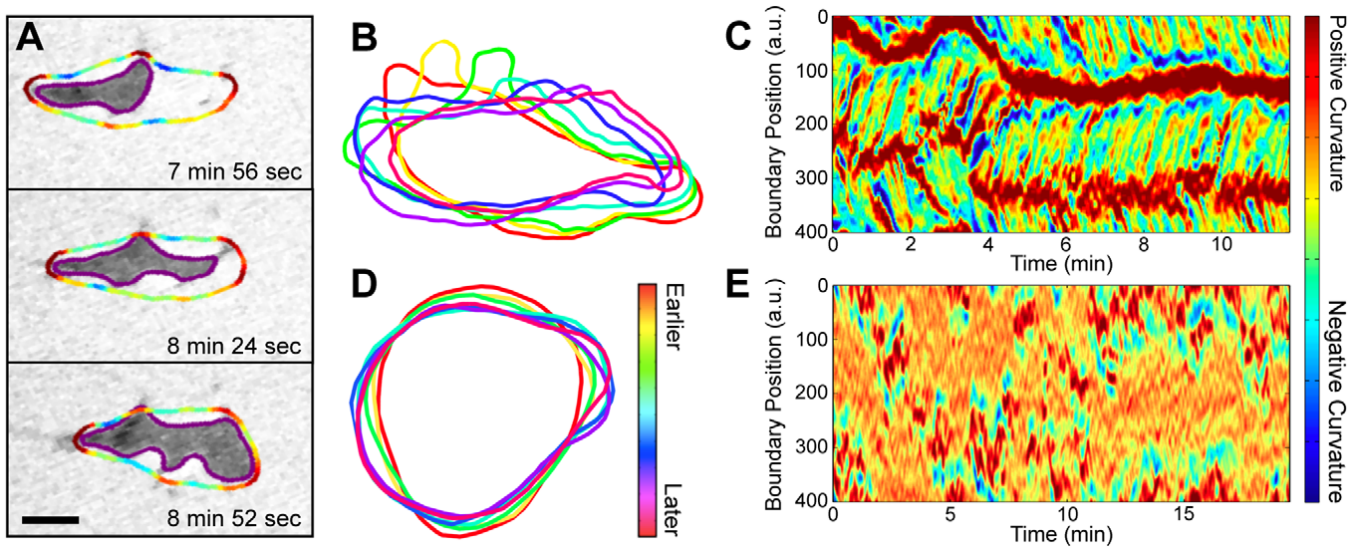
The interaction of boundary curvature waves with the substrate. (A) An IRM image sequence overlaid with the boundary of the surface contact region, shown in purple, and the boundary of the entire cell, shown colored by curvature (*video S4*). At the sides of cells, peaks in cell boundary curvature are located near peaks in surface contact region boundary curvature. Both of these curvature peaks are stationary with respect to the substrate. (Scale bar, 5 µm.) (B) The overlaid shapes of an *aca* ^−^ (non cAMP releasing) cell that is electrostatically repulsed from the substrate, and so is not adhered to the surface. The curvature peak that travels from the cell front to the cell back is moving with respect to the substrate. The centroid positions were aligned to account for drift. (4 sec. apart.) (C) The boundary curvature kymograph of this non-adherent cell (*video S5*). (D) The overlaid shapes of a non-adherent, myosin II null cell. While the cell extends regions of transient protrusive activity, it has no apparent curvature waves. (4 sec. apart.) (E) The boundary curvature kymograph of this non-adherent myosin II null cell.

Migration requires not only pseudopods but also adhesion to a surface. Thus, surface contact can indicate whether bumps on the side of the cell reflect pseudopods that have successfully adhered (and thus can contribute to motion) or unsuccessful pseudopods that failed to adhere. We imaged fluorescently-labeled, developed, WT cells, while simultaneously using internal reflection microscopy (IRM) to image the region of cell-surface contact. We extracted both the boundary of the entire cell and the boundary of the surface contact region(s) from the images. *Figure 2a* shows, for a representative cell, an IRM image sequence overlaid with the boundary of the surface contact region and the boundary of the entire cell. In this image sequence, the cell extends a protrusion that is not in contact with the surface, the protrusion makes contact with the surface, and then the area of surface contact under the protrusion grows. Our data reveal that the local protrusions at the side of the cell correspond to regions of enhanced cell-surface contact, and thus likely reflect successful pseudopods. We note that some protrusions retract quickly and never contact the surface. For example, in the movie associated with this image sequence (*video S4*), 4 out of 14 large protrusions retract before they contact the surface. Protrusions that do contact the surface are rarely retracted, indicating that contact stabilizes the protrusions.

### Cells that are not adhered to a surface exhibit traveling curvature waves

At the sides of migrating cells, regions of high curvature are in contact with the surface and remain stationary. However, in analyzing the shapes of migrating cells it is difficult to determine if regions of high curvature are stationary at the front of cells. To analyze the behavior of high curvature regions in the absence of surface contact, we analyzed the shape of *aca^−^* cells that were electostatically repelled from the surface. Since cell membranes and glass coverslips are both negatively charged, cells do not adhere to coverslips at a low salt solution [30]. The cells remain viable – upon addition of standard buffer the cells adhere to the surface and migrate. We found that for non-adherent cells, regions of high curvature actively move in a wave-like manner from the cell front to the cell back (*video S5*, *figure 2b*). *Figure 2c* shows the curvature kymograph of a non-adherent cell. Here, the curvature waves move at an average speed of 36±2 µm/min (with a standard deviation of 8 µm/min), which is much faster than the average cell speed in the movie of 8 µm/min (with a standard deviation of 5 µm/min). In both adherent and non-adherent cells, the high curvature regions tend to alternate between the left and right sides of the cell.

### Non-adhered myosin II null cells show shape oscillations instead of traveling curvature waves

We also analyzed the shape of *myoII^−^* cells that were electostatically repelled from the surface (*figures 2d,e*). Unlike *aca^−^* cells, *myoII^−^* cells do not exhibit traveling curvature waves. Instead, *myoII^−^* cells are round with localized, transient patches of protrusive activity. These protrusive patches are not confined to one region of the cell boundary, as they would be if there were a stable cell front, but rather can appear anywhere along the cell boundary.

### Cells extended over a cliff also exhibit traveling curvature waves

**Figure 3:**
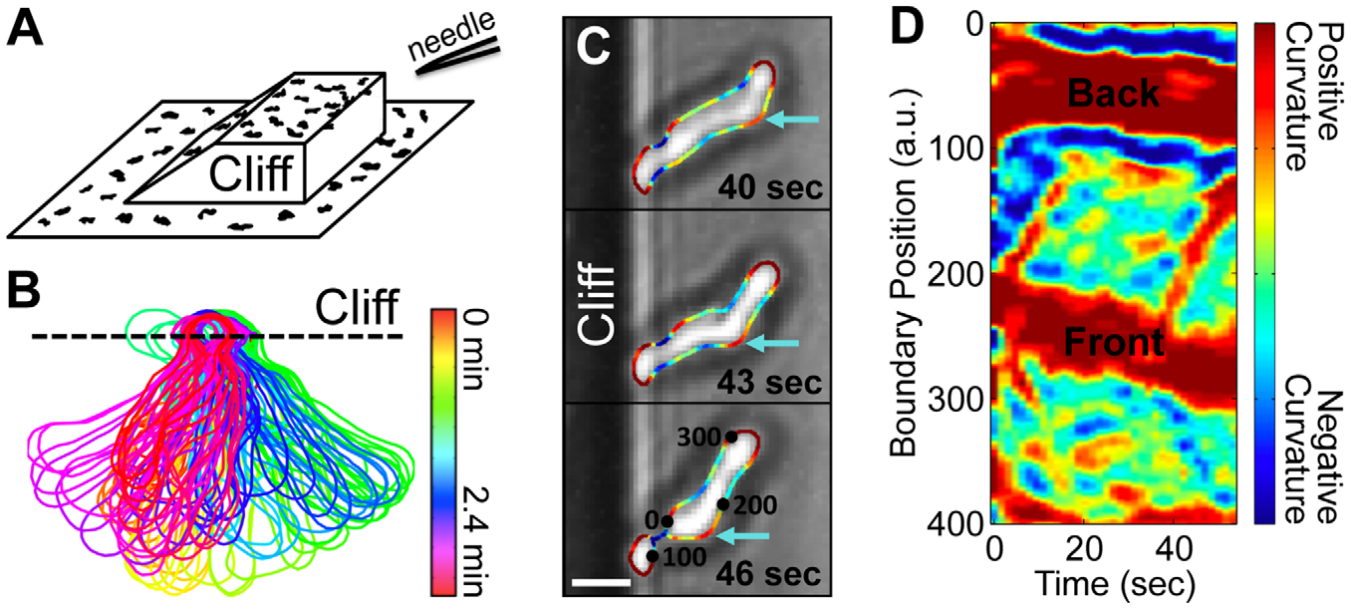
Boundary curvature waves on cells that are extended over the edge of a cliff. (A) A schematic of the 3-D surface on which cells are guided over a cliff edge. The surface closest to a cAMP-releasing needle is the cliff edge. (B) The overlaid boundaries show a cell extended over the edge of a cliff and wiggling rapidly (boundaries are 1.6 seconds apart). (C) An image sequence of a propagating curvature wave. (*video S5*). (Scale bar, 5 µm.) (D) The corresponding curvature kymograph, showing multiple curvature waves.

Using surfaces with-three dimensional topography, we also analyzed the shapes of cells that adhere only at their back, even in standard salt concentrations. To guide a cell to move away from the surface, we placed a point source of cAMP above and over the edge of a microfabricated ramp that terminated with a 75 µm tall cliff, such that the surface closest to the point source was the cliff edge. A schematic of our set-up is shown in *figure 3a*. The majority of cells that reach the edge of the cliff extend themselves over the cliff edge. Many of those cells also migrate along the cliff edge to attempt to reach the needle. We never observed a cell falling off of a cliff. Instead, cells at the cliff edge extend up to 80% of their surface area over the edge toward the cAMP source. The shape dynamics at first glance look quite distinct – the cells swing back and forth quickly over the edge (*figure 3b*). However, curvature waves are again seen in the portion of each cell that is not adhered to the cliff (which represents the majority of the cell). *Figure 3c* shows an image sequence of one such *aca^−^* cell, in which a curvature wave propagates from the front to the back of the cell (*video S6*). (This cell appeared in our previous work [31], although the boundary curvature was not analyzed.) *Figure 3d* shows the corresponding curvature kymogragh. The average curvature wave speed for the cell shown here is 29±3 µm/min (as calculated from 9 waves over 2.4 minutes). The average curvature wave speed of cells extended over the edge of a cliff is comparable to the curvature wave speed of cells that are electrostatically repelled from their substrate. The swinging of the cell appears correlated with curvature waves hitting the surface, which provides a simple mechanism for cellular reorientation, and hence the exploration of 3D space.

### Curvature peaks are seen only after cell polarization

**Figure 4:**
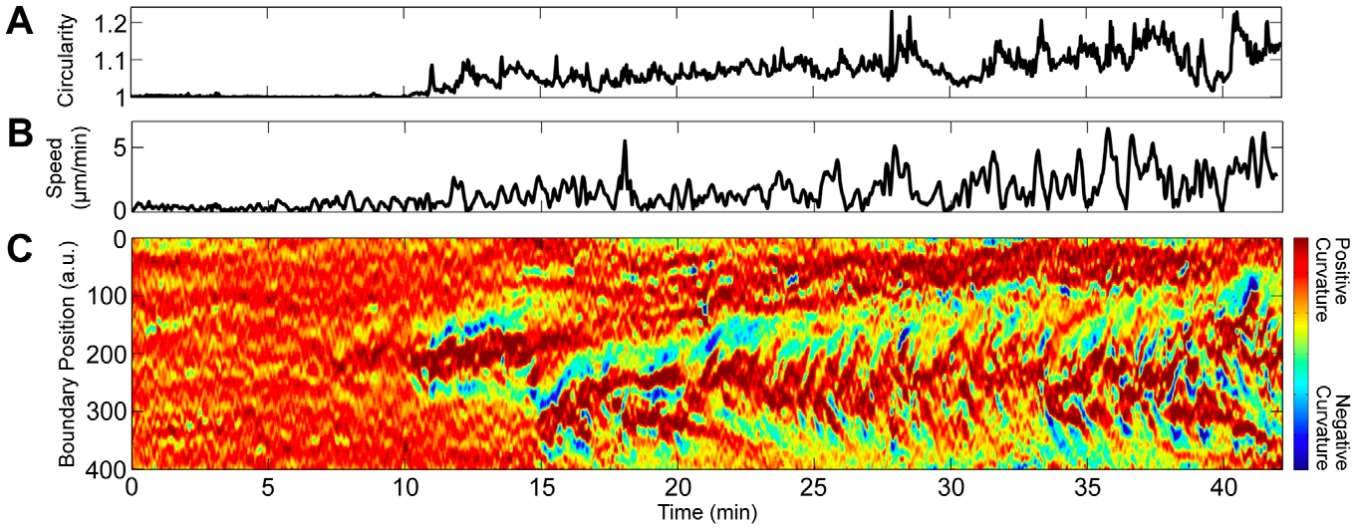
Polarity symmetry breaking of an *aca* ^−^ (non cAMP releasing) cell. (A) During polarization, non-circularity, the normalized ratio of perimeter to the perimeter of a circle with the same area, increases in an oscillatory fashion. (B) The speed of the cell centroid. (C) Boundary curvature, which prior to polarization is mostly static, begins exhibiting organized curvature waves (*video S7*).

To explore the onset of curvature peaks at the sides of cells, we analyzed the dynamic shape of polarizing *Dictyostelium*. Cells are almost always round when initially placed on a surface, although WT cells quickly polarize and begin to migrate. *Aca^−^* cells, which do not produce cAMP, are more basal (quiescent) than WT cells. However, WT and *aca^−^* cells migrate with comparable speed and directional persistence [8], and exhibit indistinguishable curvature peaks (*figure S2a*). We therefore analyzed the onset of curvature peaks in *aca^−^* cells. Even prior to polarization, cells send out small, quickly retracted protrusions. Since these transient protrusions are more visible in phase-contrast images than in images of fluorescently dyed cells, we also analyzed phase-contrast movies (*figure* 4). The polarization of a fluorescently dyed *aca^−^* cell is shown in *figure S2a (video S8)*.

When a cell polarizes, its shape elongates. To quantify the degree of polarization, we define the non-circularity as the ratio of the cell perimeter to its area, normalized so that the non-circularity of a circle is 1. The non-circularity, centroid velocity, and boundary curvature of a polarizing *aca^−^* cell are shown in *figure 4*. While the non-circularity is initially near one, it soon begins to increase in an oscillatory fashion. The boundary curvature, on the other hand, changes pattern abruptly at the onset of motion. While the curvature prior to polarization is slightly irregular, curvature peaks (tilted red lines in *figure 4c*) appear only after polarization. Hence, polarization and migration coincide with a change in the nature of protrusions from being localized and intermittent to being more continuous and possibly wave-like.

### At the front of adherent cells, curvature peaks are associated with boundary motion

**Figure 5:**
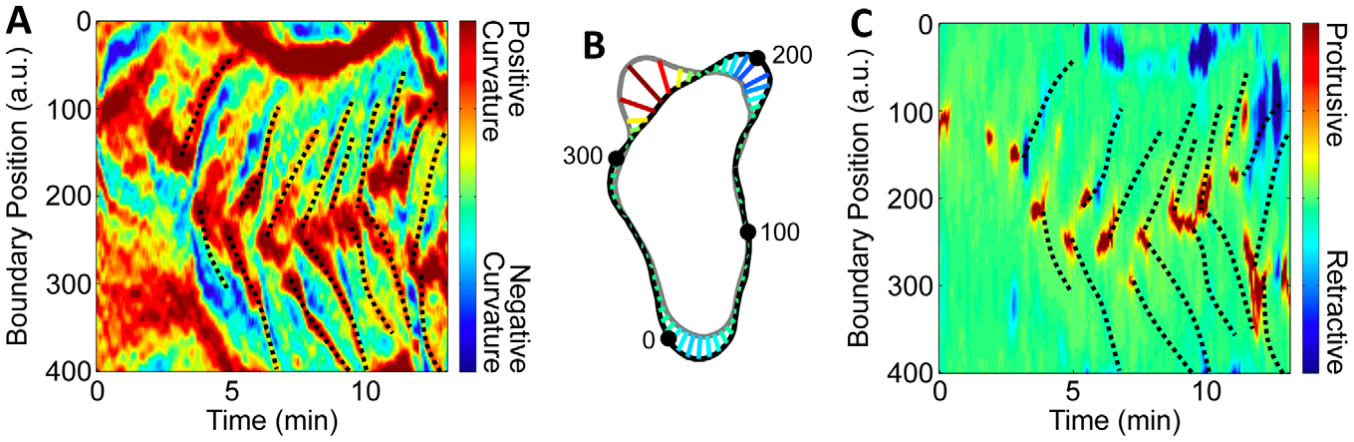
The initialization of curvature waves is associated with protrusive motion. (A) The boundary curvature kymograph of the cell shown in *figure 2a*. Curvature waves, shown as dashed black lines, are drawn on. (B) One measure of local boundary speed is the magnitude of the motion mapping. Here, the cell shape at 6 minutes is shown as the black boundary, while the shape 12 seconds later is the gray boundary. The motion mapping vectors, shown here colored by magnitude (colormap as in *c*), connect the boundary points in the earlier frame to boundary points in the later frame. Only every eighth mapping vector is shown. (C) The local motion kymograph with the overlaid position of the curvature waves, which appear as dashed black lines. Protrusive events usually coincide with the initialization of curvature waves.

Curvature peaks are suggestive of protrusions, because a localized protrusion is necessarily associated with a localized region of high curvature. To compare boundary curvature to motion, we developed a measure of local boundary motion. We calculated the motion of each boundary point by measuring the distance to the closest boundary point in a later frame and then smoothing over the list of mapped to boundary points. Protrusive motion was defined to be positive, while retractive motion was defined to be negative. *Figure 5b* shows representative local motion mapping vectors colored by the value of the local motion measure, while *figure 5c* shows the local boundary motion kymograph of the same developed, WT cell shown in *figure 2a*, (See *figures S1b* and *S2b* for additional plots of WT and *aca^−^* cells.) From local motion kymographs, we see that cells have two regions of activity, one associated with protrusions and one with retractions. Neither the fronts nor the backs of cells move at constant speed; rather, they start and stop intermittently. However, while the location along the boundary of retractions shifts little from retraction to retraction, protrusions tend to zig-zag.

We can compare boundary curvature to boundary motion by comparing the curvature and local motion kymographs. Curvature peaks (shown as dashed black lines) are overlaid on *figures 5a and 5c*. In general, we find that the initiation of curvature peaks tends to coincide with the growth of protrusions, at the front of the cell. (Also, *figure S4* shows how these curvature peaks relate to the distance from the cellular boundary to the footprint.) Thus protrusive motion might travel along the boundary in the same manner as the curvature peaks.

### On short time-scales, protrusive motion travels ballistically along the cell boundary

**Figure 6:**
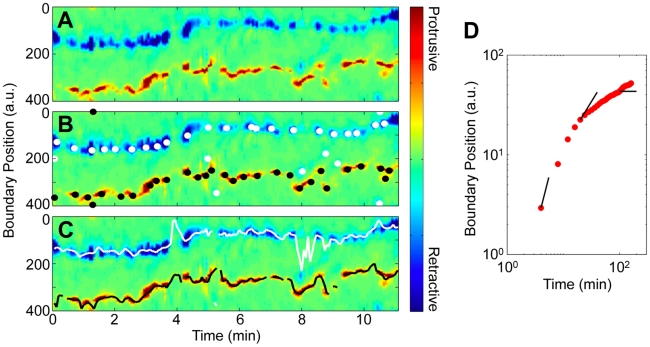
Boundary motion can be analyzed as a series of discrete protrusive and retractive events or as continuous movement. (A) In this plot of the evolution of local boundary motion, protrusive motion appears red, while retractive motion appears blue (*video S9*). (B) Extracted individual protrusions are shown as black dots, while extracted retractions are shown as white dots. (C) The averaged location of protrusive and retractive motion, which is defined in each frame where boundary motion is above a noise threshold, are shown here. This cell is also shown in *figure 1*. (D) The mean squared displacement of the mean protrusive location is ballistic on short time-scales. The black lines, from left to right, have slopes of 2, 1 and 0 micrometers/min.

Protrusive motion has often been discretized into pseudopod extension and retraction events. Here we analyze protrusive motion both with and without discretization and show that discretization may hide the wave-like nature of the protrusive process. We first analyze boundary motion under the assumption that protrusions and retractions are discrete events. The times and locations of individual protrusions and retractions along the boundary were defined as the peaks and valleys of the local motion measure, respectively. *Figure 6b* shows the protrusions and retractions extracted from the local motion data shown in *figure 6a*. Protrusions are shown as black dots, while retractions are shown as white dots. As expected based on the visualization in *figure 6a*, protrusions are more spread out at the front of the cell than are retractions at the back. However, some retractions do occur at the front of the cell when protrusions are retracted. We calculated temporal and spatial statistics based on data from 26 self-aggregating WT cells, including those with developmental times 1.5 hours longer or shorter than our normally used time. We found a total of 2219 protrusions and 2220 retractions in these data. On average, WT cells exhibit 2.9±0.2 protrusions/minute and 2.9±0.2 retractions/minute. Boosgraaf *et al.* found a similar frequency of 4 protrusions/minute [17]. The statistics of the extracted protrusions and retractions are further explored in [29].

While analyzing protrusions as discrete events yields results consistent with prior work [17], two facets of the kymograph of local protrusions and retractions in *figure 6a* indicate that this discretization can mask continuous or wave-like characteristics of protrusive motion. First, protrusions are not well separated from each other. Second, many protrusions appear as tilted streaks in the kymograph, indicating that lateral motion occurs during the protrusion.

To analyze protrusive and retractive motion as continuous boundary movement, we define the location of greatest protrusion and retraction activity for each frame as the location of the weighted average of the protrusive or retractive motion. *Figure 6c* shows a representative example of extracted mean protrusion and retraction locations.

We measured the mean squared displacement (MSD) of the average protrusion location along the boundary (*figures 6d* *and S3*). Note that this is not the MSD of centroid motion, but the MSD of protrusive motion along the boundary of the cell. We find that on short time scales, the protrusive motion along the boundary is nearly ballistic. As the time scale increases, protrusive motion becomes caged to the front of the cell. The transition between the time scales for ballistic and caged motion occurs at roughly 20 seconds, which corresponds to the average frequency of 2.9±0.2 protrusions/minute derived from discrete protrusions. Ballistic motion on short time scales suggests that peak protrusive activity travels along the boundary in a wave-like manner, similar to the peaks in boundary curvature. We estimate from the MSD at 8 seconds that the speed of these waves is at least 21 µm/min. This estimate is likely to be less than the true wave speed, since local motion (from which the mean protrusion is found) is measured across frames that are obtained 12 seconds apart, and since the displacement is not completely ballistic from 0 to 8 seconds.

Together, these findings indicate that for adherent cells, protrusive activity is continuous and constantly shifts along the leading edge of the cell in a wave-like manner similar to the dynamics of the wave-like high curvature regions observed both in suspended cells and in cells extended over cliff edges.

## Discussion

Using quantitative analysis of cell shape dynamics, we have demonstrated the existence of wave-like characteristics in the local shape of *Dictyostelium discoideum* and have elucidated their likely role in cell migration.

In order to track the local boundary from frame to frame, we maintained a constant number of boundary points per frame. This approach allows for a robust, 1∶1 mapping between points in subsequent frames (see SI). Other recent approaches in which the lengths of boundary segments are maintained [32] align the boundary at one predetermined point, and so are most informative only near that point. With a constant number of boundary points per frame, the evolution of the local curvature and local protrusions (or any other metric associated with each boundary point) can be analyzed readily. A constant number of boundary points also makes possible the simple visual representation of shape dynamics through kymographs, which allows us to represent a movie of a migrating cell in a single image [29]. We have two major findings:

### 

#### (1) The local shape of non-adherent *Dictyostelium discoideum* exhibits traveling curvature waves

Kymographs of boundary curvature reveal regions of high curvature that start at the leading edge of the cell and travel backward along alternating sides of the cell at an approximately constant speed of ∼35 µm/min. Such curvature waves are prominent in cells that are not adherent to the surface (*figure 2*), and in cells that are extending over the edge of a cliff (f*igure 3*), but are not present in non-adhered *myoII^−^* cells. The curvature waves thus may be associated with acto-myosin dynamics, either actin polymerization waves, which have been found in migrating *Dictyostelium* cells [23] and have been observed in other cells such as neutrophils [27], or with dynamic myosin contractility.

#### (2) The location of protrusive activity shifts continuously and coincides with the source of curvature peaks

Using a measure of local motion, we determined that the locations of protrusive activity are associated with curvature peaks at the fronts of cells (*figure 5*). In previous studies, protrusions were treated as discrete activities of the cell [28]. Some of these studies found that new protrusions often split off from existing protrusions [17], and recent data, which were analyzed by skeletonizing the cell, showed that protrusions can move toward the cell back [18]. Other studies found that the direction of the chemotactic signal affects the retractions of existing pseudopods, but not the pattern of generation of new pseudopods [28]. Our results show that treating protrusive motion as a continuous process rather than as a series of discrete events provides more detailed information. Instead of trying to identify individual protrusions, which requires making potentially artificial distinctions among protrusions, we analyze protrusive motion quantitatively as a continuous process, which reveals its fundamentally wave-like character. On short time scales, the location of strongest protrusive activity is not stationary, but shifts along the leading edge of the cell at a speed of at least 21 µm/min for 20 seconds on average.

Local protrusive motion at the cell front transitions from ballistic to caged at about 20 seconds. This 20 second characteristic time is consistent with the finding of Meili *et al.* that, on average, protrusions are extended for 22 seconds before they exert a force on the surface [33]. Thus, our findings are consistent with waves of protrusive activity that stop moving when they contact the surface, as observed for neutrophils [27]. However, although localized protrusions stop moving relative to the surface, they remain visible as high curvature regions. Curvature peaks then provide insights into the history of protrusive activity, and allow us to compare chemokinesis, chemotaxis, and the motion of non-adherent cells.

Indeed, we find the same results both for self-aggregating WT cells and for chemokinesing *aca^−^* cells. Chemokinesing cells also have ballistically-traveling curvature waves and zig-zagging protrusions (see *figure S2*). A group of self-aggregating WT cells can be distinguished from a group of chemokinesing *aca^−^* cells by their distinct long-term directionality. However, the kymographs of local curvature and local protrusions are not distinguishable. We also present evidence that similar wave processes govern the shape and shape dynamics of both adherent and non-adherent cells. In particular, the kymographs of local curvature are similar for both types of cells (see *figure 1* for adherent cells, compared to *figure 2* for non-adherent cells, and *figure 3* for cells at the edge of cliffs).

The similarities in protrusive activity and shape dynamics of chemotaxing, chemokinesing, and suspended cells is hard to reconcile with models in which pseudopods are triggered directly by directional chemical signals. Our results are consistent with recent models that treat the cellular migratory apparatus as an excitable system [34], since wave-like and oscillatory dynamics are common in excitable systems.

### We hypothesize that wave-like protrusive activity may have three main consequences:

1. Wave-like protrusions provide a simple and robust mechanism for directed migration. Chemotactic signals are not needed to trigger migration, since protrusive activity is self-sustaining. This view is consistent with observations that cells continue to migrate for hours after a temporary migratory signal [8] without further stimulus and with speed and directional persistence comparable to those of chemotaxing cells. Chemotactic signals merely need to tune the excitability to steer cells [34].
2. Wave-like protrusions may allow cells to migrate in a viscous environment. Recent reports indicate that both *Dictyostelium* and neutrophils can swim in fluids that are significantly more viscous than water [35]. Front extension and back retraction, one standard simplified view of amoeboid and neutrophil cell migration, cannot enable migration through a viscous fluid. The traveling protrusive waves observed here, on the other hand, break symmetry and so could explain the ability of cells to swim.
3. Wave-like protrusions may allow cells to search for surfaces in 3D environments. Away from a surface, a protrusion that advects backwards along the edge of the cell seems to lead to a wiggling motion (*figure 3*). This wiggling could in turn act as a search mechanism for surfaces for adhesion. Thus, zig-zag motion, the dominant mode of fast amoeboid migration on a flat surface, may be a side effect of a wave-like migration mechanism that is suitable for both migrating on surfaces and swimming.

## Materials and Methods

### Cell culture and imaging

*Dictyostelium discoideum* cells, WT (AX3), adenylyl cyclase A null, and myosin II null (both *aca^−^* and *myoII^−^* are in an AX3 background) were prepared as described previously [8]. Unless otherwise specified, WT and *aca^−^* cells were developed for 5 hours. *MyoII^−^* cells were developed for 6 hours. Since *myoII^−^* cells do not divide in suspension culture, they were harvested directly from plate cultures. In electrostatic repulsion experiments, cells were washed and run in 10^−3^ diluted phosphate buffer (see [8]). All cells, except in cliff experiments, were cytoplasmically dyed with CellTracker Green CDMFA (Invitrogen) [8]. Acrylic resin micro-cliffs were fabricated using multiphoton absorption polymerization [36] (See text S1 for more information). For cliff experiments, phase-contrast images were obtained with a 10× objective every 1.55 seconds. For footprint and polarization experiments, fluorescence, phase contrast and internal reflection microscopy (IRM) images were captured with a 40× objective every 2 or 4 seconds. For electrostatic repulsion experiments, fluorescence images were obtained on a Leica TCS SP2 confocal microscope with a 100× objective every 2 seconds. For the remaining experiments, fluorescence images were obtained on the same confocal microscope with a 40× objective every 4 seconds.

### Tracking local boundary motion

Image sequences that show the migration of individual cells were pre-processed using ImageJ to enhance contrast and to remove other cells. The shape of the cell was then extracted using a snake algorithm. We adapted sample code [37] to follow the shape of a cell automatically throughout an image sequence and to parametrize the shape with 400 boundary points. Note that while within each frame neighboring points are equidistant along the perimeter, the distance between neighboring points varies across frames as the length of the cell perimeter changes.

With a constant number of boundary points per frame, *all* local boundary points can be tracked simultaneously by defining a 1∶1 mapping between the points in each frame and the successive frame. We chose the mapping that maintained the numbering sequence of boundary points, and that minimized the sum of the square distances points moved from frame to frame. An example of such a tracking mapping is shown in *video S10*. (See text S1 for more about tracking.)

### Calculating boundary point associated measures

At each boundary point, we calculate the boundary curvature by fitting a circle to that boundary point and the two points that are 10 boundary points away from it. The magnitude of the boundary curvature is then defined as the reciprocal of the radius of that circle. If the midpoint of the two points 10 boundary points away is inside the cell, the curvature is defined as positive, otherwise it is defined as negative. For visualization, the curvature is smoothed over 3 boundary points and 3 frames, and the color scale is cut off at a maximum curvature magnitude.

To calculate local motion, we first mapped each boundary point to the closest boundary point in the frame 12 seconds later. This time interval was chosen so that boundary motion dominates over image noise. Next, we used an averaging window to smooth the mapping twice. (The first smoothing had a window size of 19 boundary points, while the second had a size of 15 boundary points.) *Figures S5a* and *b* show an example of the mapping before and after smoothing. We defined local motion as the distance between mapped points. Like the curvature, when visualized, the color scale for local motion is cut off at a maximum magnitude. (See text S1 for more information.)

## Supporting Information

Figure S1 The (A) boundary curvature and (B) local motion kymographs of a self-aggregating, wild-type cell (*video S3*).
(TIF)

Figure S2 The polarization of a fluorescently dyed *aca^−^* cell (*video S8*). The (A) boundary curvature and (B) local motion kymographs are similar to those of wild-type cells.
(TIF)

Figure S3 Distributions of the magnitude of mean protrusion displacements along the boundary. The durations of the displacements vary from 4 to 160 seconds and are 4 seconds apart.
(TIF)

Figure S4 Curvature waves are visible in the cellular footprint (*video S4*). The distance between the cell boundary and the cell footprint overlaid by the cell's curvature waves, shown as white dashed lines. This is the same cell shown in *figures 2a* and *5*.
(TIF)

Figure S5 The local motion mapping. (A) First, each boundary point in a frame is mapped to the closest boundary point in the frame 12 seconds later. A representative frame's boundary is shown as a solid line, the boundary in the frame 12 seconds later by a dashed line, and the mapping between boundary points by blue lines. (B) Next, we smooth over target boundary points, pulling mapping vectors into protrusions, and more evenly distributing vectors in retractions. The magnitude of these blue mapping vectors is then defined as the magnitude of our local motion measure.
(TIF)

Text S1 Supplemental materials and methods.
(PDF)

Video S1 A migrating, wild-type *Dictyostelium* cell. The boundary is colored by curvature (see *figure 1*). (Scale bar, 5 µm; Duration, 22 minutes.)
(MOV)

Video S2 A migrating, wild-type cell. Colored markers indicate the position of every 50^th^ boundary point. (There are 400 total boundary points.) The red dot represents boundary point 0, the orange dot boundary point 50, etc. (see *figure 1*). (Scale bar, 5 µm; Duration, 22 minutes.)
(MOV)

Video S3 An additional, migrating, wild-type cell. The boundary is colored by curvature (see *figure S1*). (Scale bar, 5 µm; Duration, 40 minutes.)
(MOV)

Video S4 Curvature waves in the boundary, shown colored by curvature, and footprint, shown in purple, of a migrating cell (see *figures 2a* *and* *5*). (Scale bar, 5 µm; Duration, 13 minutes.)
(MOV)

Video S5 *Aca^−^* cells moving in a very dilute salt solution. Curvature waves that travel with respect to the surface can be seen. (see *figures 2b–d*). (Scale bar, 20 µm; Duration, 15.4 minutes.)
(MOV)

Video S6 Curvature waves in a cell extended over the edge of a cliff (see *figure 3*). (Scale bar, 5 µm; Duration, 2.4 minutes.)
(MOV)

Video S7 The polarization of an *aca^−^* cell. The boundary, shown colored by curvature, has curvature waves only after polarization (see *figure 4*). (Scale bar, 5 µm; Duration, 43 minutes.)
(MOV)

Video S8 The polarization of an additional *aca^−^* cell. The boundary, shown colored by curvature, has curvature waves only after polarization (see *figure S2*). (Scale bar, 5 µm; Duration, 45 minutes.)
(MOV)

Video S9 The local motion measure. Each frame's boundary is shown as a solid line, the boundary in the frame 12 seconds later as a dashed line, and every 6^th^ tracking mapping vector as a line colored by the value of the local motion measure (see *figures 1* *and* *6a–c*). (Scale bar, 5 µm; Duration, 22 minutes.)
(MOV)

Video S10 Tracking the local boundary of a migrating, wild-type cell. Each frame's boundary is shown as a solid line, the boundary in the next frame as a dashed line, and every 20^th^ tracking mapping vector as a red line (see *figure 1*). (Scale bar, 5 µm; Duration, 22 minutes.)
(MOV)

## References

[1] Wagle MA, Tranquillo RT (2000). A self-consistent cell flux expression for simultaneous chemotaxis and contact guidance in tissues.. J Math Biol.

[2] Yang X, Dormann D, Munsterberg AE, Weijer CJ (2002). Cell movement patterns during gastrulation in the chick are controlled by positive and negative chemotaxis mediated by FGF4 and FGF8.. Dev Cell.

[3] Capogrosso Sansone B, Scalerandi M, Condat CA (2001). Emergence of taxis and synergy in angiogenesis.. Phys Rev Lett.

[4] Kassis J, Lauffenburger DA, Turner T, Wells A (2001). Tumor invasion as dysregulated cell motility.. Semin Cancer Biol.

[5] Bagorda A, Parent CA (2008). Eukaryotic chemotaxis at a glance.. J Cell Sci.

[6] Friedl P (2004). Prespecification and plasticity: shifting mechanisms of cell migration.. Curr Opin Cell Biol.

[7] Parent CA, Devreotes PN (1999). A cell's sense of direction.. Science.

[8] McCann C, Kriebel P, Parent C, Losert W (2010). Cell speed, persistence and information transmission during signal relay and collective migration.. J Cell Sci.

[9] Kriebel PW, Barr VA, Parent CA (2003). Adenylyl cyclase localization regulates streaming during chemotaxis.. Cell.

[10] Rericha EC, Parent CA (2008). Steering in quadruplet: the complex signaling pathways directing chemotaxis.. Sci Signal.

[11] Rickert P, Weiner OD, Wang F, Bourne HR, Servant G (2000). Leukocytes navigate by compass: roles of PI3Kgamma and its lipid products.. Trends Cell Biol.

[12] Van Haastert PJM, Bosgraaf L (2009). The local cell curvature guides pseudopodia towards chemoattractants.. HFSP J.

[13] King JS, Insall RH (2009). Chemotaxis: finding the way forward with Dictyostelium.. Trends Cell Biol.

[14] Machacek M, Danuser G (2006). Morphodynamic profiling of protrusion phenotypes.. Biophys J.

[15] Maeda YT, Inose J, Matsuo MY, Iwaya S, Sano M (2008). Ordered patterns of cell shape and orientational correlation during spontaneous cell migration.. PLoS One.

[16] Soll DR, Wessels D, Kuhl S, Lusche DF (2009). How a Cell Crawls and the Role of Cortical Myosin II.. Eukaryot Cell.

[17] Bosgraaf L, Van Haastert PJM (2009). The Ordered Extension of Pseudopodia by Amoeboid Cells in the Absence of External Cues.. PLoS ONE.

[18] Xiong Y, Kabacoff C, Franca-Koh J, Devreotes P, Robinson D (2010). Automated characterization of cell shape changes during amoeboid motility by skeletonization.. BMC Syst Biol.

[19] Döbereiner H-G, Dubin-Thaler BJ, Hofman JM, Xenias HS, Sims TN (2006). Lateral Membrane Waves Constitute a Universal Dynamic Pattern of Motile Cells.. Phys Rev Lett.

[20] Xiong Y, Huang CH, Iglesias PA, Devreotes PN (2010). Cells navigate with a local-excitation, global-inhibition-biased excitable network.. Proc Natl Acad Sci U S A.

[21] Blanchard GB, Murugesu S, Adams RJ, Martinez-Arias A, Gorfinkiel N (2010). Cytoskeletal dynamics and supracellular organisation of cell shape fluctuations during dorsal closure.. Development.

[22] Killich T, Plath PJ, Wei X, Bultmann H, Rensing L (1993). The locomotion, shape and pseudopodial dynamics of unstimulated Dictyostelium cells are not random.. J Cell Sci.

[23] Vicker MG (2000). Reaction-diffusion waves of actin filament polymerization/depolymerization in Dictyostelium pseudopodium extension and cell locomotion.. Biophys Chem.

[24] Vicker MG (2002). Eukaryotic cell locomotion depends on the propagation of self-organized reaction-diffusion waves and oscillations of actin filament assembly.. Exp Cell Res.

[25] Gerisch G, Bretschneider T, Muller-Taubenberger A, Simmeth E, Ecke M (2004). Mobile Actin Clusters and Traveling Waves in Cells Recovering from Actin Depolymerization.. Biophys J.

[26] Bretschneider T, Anderson K, Ecke M, Muller-Taubenberger A, Schroth-Diez B (2009). The Three-Dimensional Dynamics of Actin Waves, a Model of Cytoskeletal Self-Organization.. Biophys J.

[27] Weiner OD, Marganski WA, Wu LF, Altschuler SJ, Kirschner MW (2007). An Actin-Based Wave Generator Organizes Cell Motility.. PLoS Biol.

[28] Andrew N, Insall RH (2007). Chemotaxis in shallow gradients is mediated independently of PtdIns 3-kinase by biased choices between random protrusions.. Nat Cell Biol.

[29] Driscoll MK, Fourkas JT, Losert W (2011). Local and global measures of shape dynamics.. Phys Biol.

[30] Socol M, Lefrou C, Bruckert F, Delabouglise D, Weidenhaupt M (2010). Synchronization of Dictyostelium discoideum adhesion and spreading using electrostatic forces.. Bioelectrochemistry.

[31] Driscoll M, Kopace R, Li L, McCann C, Watts J (2009). The Adventures of Dicty, the Dictyostelium cell.. Chaos.

[32] Bosgraaf L, Van Haastert PJ (2009). Quimp3, an automated pseudopod-tracking algorithm.. Cell Adh Migr.

[33] Meili R, Alonso-Latorre B, del Alamo JC, Firtel RA, Lasheras JC (2009). Myosin II is essential for the spatiotemporal organization of traction forces during cell motility.. Mol Biol Cell.

[34] Xiong YA, Huang CH, Iglesias PA, Devreotes PN (2010). Cells navigate with a local-excitation, global-inhibition-biased excitable network.. Proc Natl Acad Sci U S A.

[35] Barry NP, Bretscher MS (2010). Dictyostelium amoebae and neutrophils can swim.. Proc Natl Acad Sci U S A.

[36] LaFratta CN, Fourkas JT, Baldacchini T, Farrer RA (2007). Multiphoton fabrication.. Angewandte Chemie.

[37] Xu C, Prince JL (1998). Snakes, shapes, and gradient vector flow.. IEEE Trans Image Process.

