# Supplementary figures and images for: Cell Shape Dynamics: From Waves to Migration
Source: PLoS Comput Biol. 2012 Mar 15;8(3):e1002392. doi: 10.1371/journal.pcbi.1002392 (PMC3305346; doi:10.1371/journal.pcbi.1002392)

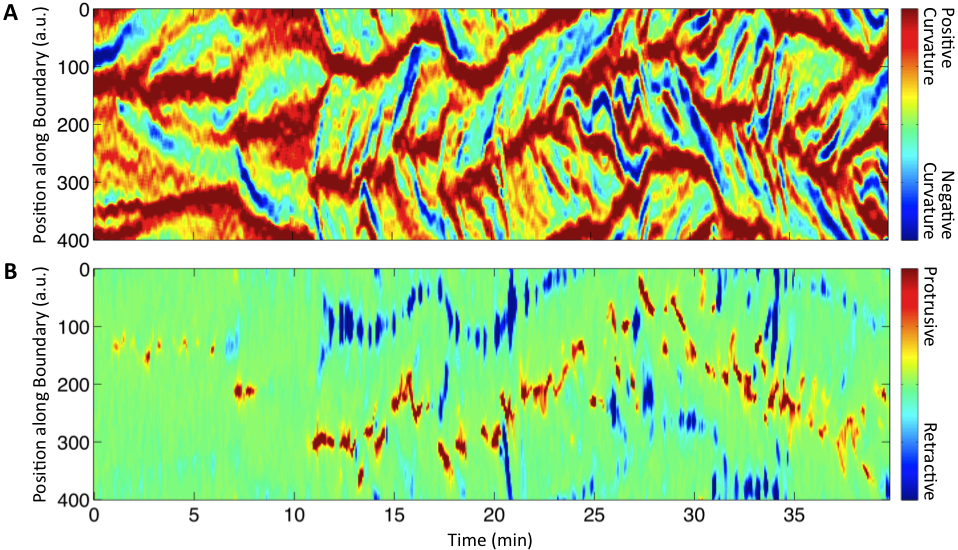

Supplement: Figure S1 — The (A) boundary curvature and (B) local motion kymographs of a self-aggregating, wild-type cell (video S3). (TIF) [file pcbi.1002392.s001.tif]

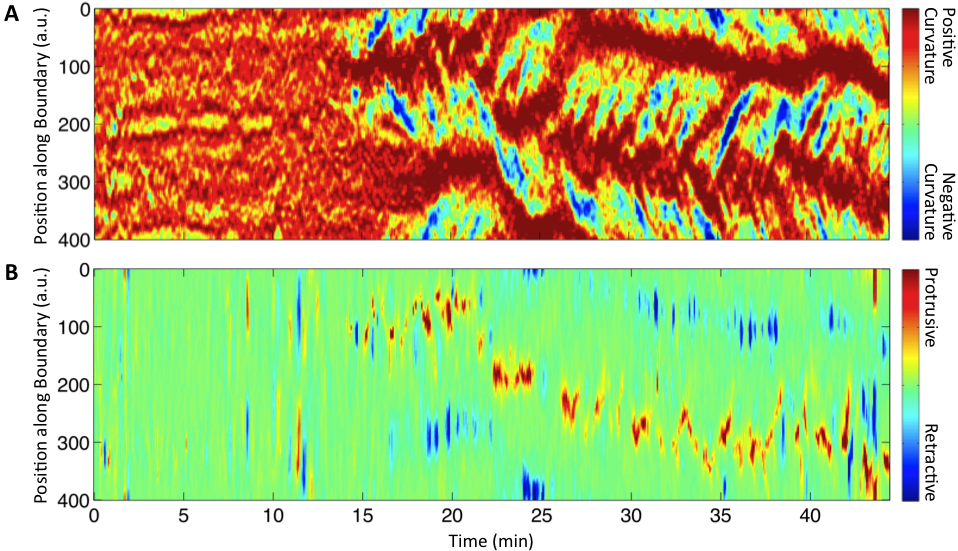

Supplement: Figure S2 — The polarization of a fluorescently dyed aca− cell (video S8). The (A) boundary curvature and (B) local motion kymographs are similar to those of wild-type cells. (TIF) [file pcbi.1002392.s002.tif]

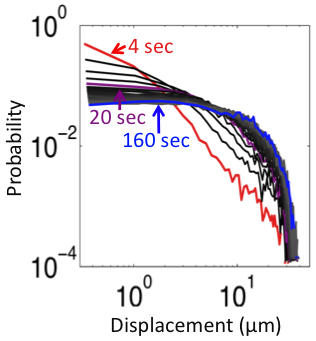

Supplement: Figure S3 — Distributions of the magnitude of mean protrusion displacements along the boundary. The durations of the displacements vary from 4 to 160 seconds and are 4 seconds apart. (TIF) [file pcbi.1002392.s003.tif]

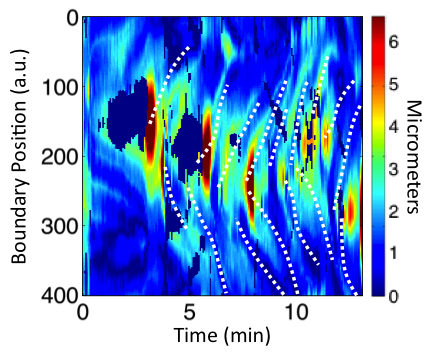

Supplement: Figure S4 — Curvature waves are visible in the cellular footprint (video S4). The distance between the cell boundary and the cell footprint overlaid by the cell's curvature waves, shown as white dashed lines. This is the same cell shown in figures 2a and 5 . (TIF) [file pcbi.1002392.s004.tif]

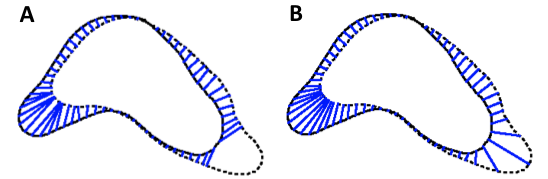

Supplement: Figure S5 — The local motion mapping. (A) First, each boundary point in a frame is mapped to the closest boundary point in the frame 12 seconds later. A representative frame's boundary is shown as a solid line, the boundary in the frame 12 seconds later by a dashed line, and the mapping between boundary points by blue lines. (B) Next, we smooth over target boundary points, pulling mapping vectors into protrusions, and more evenly distributing vectors in retractions. The magnitude of these blue mapping vectors is then defined as the magnitude of our local motion measure. (TIF) [file pcbi.1002392.s005.tif]
