# Supplementary material for: Cell Shape Dynamics: From Waves to Migration
Source: PLoS Comput Biol. 2012 Mar 15;8(3):e1002392. doi: 10.1371/journal.pcbi.1002392 (PMC3305346; doi:10.1371/journal.pcbi.1002392)
Supplement: Text S1 — Supplemental materials and methods. (PDF) [file pcbi.1002392.s006.pdf]

Supplemental Materials and Methods for
Cell Shape Dynamics: From Waves to Migration

Cliff Fabrication

Micro-cliffs were fabricated using multiphoton absorption polymerization (MAP) [1]. The prepolymer resin was composed of 54 wt % dipentaerythritol pentaacrylate (Sartomer), 43 wt % tris(2-hydroxyethyl) isocyanurate triacrylate (Sartomer), and 3 wt % Lucirin TPO-L photoinitiator (BASF). The resin was polymerized using a Ti:sapphire laser (Coherent Mira 900-F) that produced 775 nm, 100 fs pulses at 76 MHz. A 20× objective was used to direct a 17 mW beam into the resin. Using a stage to move the resin through the laser's focal point, 3-D structures were fabricated by polymerizing multiple, closely-spaced lines. The finished cliffs were flat-topped ramps 50 μm tall and 300 μm long (see *figure 3a*). The horizontal, plateau top and the sloped ramp face were both 100 μm wide, and shared a 300 μm long edge. The cliff was fabricated by polymerizing 300 μm long parallel lines that were 0.75 μm apart horizontally and 2.5 μm apart vertically. Lines on the top of the ramp (both the plateau and the sloped face) were fabricated with a stage speed of 50 μm/sec, while all other lines were fabricated at a speed of 150 μm/sec. Unpolymerized resin was removed with two, successive, five minute dimethylformamide washes, followed by two, five minute ethanol washes. Each ramp was then replicated with a polydimethylsiloxane mold, as described previously [2]. The prepolymer resin described above was cured in the mold for 10 minutes under a UV lamp, the mold was removed, and then the ramp was baked for 10 minutes at 110° C. We previously found that the speed of Dictyostelium on this cured resin is similar to its speed on glass [3].

Image Sequence Pre-processing

Individual cells were selected for analysis, and their image sequences were pre-processed using ImageJ (NIH). Any feature that might make automatic extraction of the contour via snake algorithms difficult, such as a nearby cell, or materials shed by the cell, was painted the background color. Image contrast and brightness were then enhanced until the cell interior was white, the cell exterior black, and the cell edge varying shades of gray. (IRM image sequences with dark features on a bright background were first inverted.) Each sequence was then despeckled to remove noise, and the gradient was subsequently taken. Phase contrast images of polarizing cells were nearly binarized by adjusting the brightness and contrast until the cell interior and exterior were mostly white, and the cell edge mostly black. These images were not despeckled, and the gradient was not taken.

Snake Algorithm Parameters

Existing Matlab code that demonstrates the snake algorithm was modified as described in Materials and Methods. The unnormalized, gradient vector field option was used to extract boundaries. We set alpha (the tension) to 0.0002, beta (the rigidity) to 0.001, gamma (the step size) to 1, and kappa (the external force weight)

to 0.6. The code was further modified so that output boundary point indices always increased counter-clockwise.

Tracking and Motion Mappings

The snake algorithm outputs cell shapes as a list of boundary points. Tracking the local boundary then requires mapping the boundary points in each frame to the boundary points in the next frame. This procedure will be called the "Tracking Mapping". For ease of visualization and quantification, we would like to have a constant number of boundary points per frame, and we would like to track all of the boundary points from frame to frame. We therefore need to define a 1:1 tracking mapping. Mappings with crossing mapping vectors can be excluded, since these crossings are topological violations [4]. (For instance, mapping point 1 in frame A to point 2 in frame B and point 2 in frame A to point 1 in frame B is a topological violation.) Thus the only possible remaining 1:1 mappings are rotations of one another. Among those, we choose the mapping that introduces the least rotation by minimizing the sum of the square distances between mapped points.

The magnitude of the tracking mapping is a measure of global boundary point motion. However, this measure is not well suited to protrusive motion, since a single protrusion results in the measured movement of every boundary point. Instead, we would like a measure that finds motion in only the boundary points that comprise the protrusion. We then need a local measure that is not based on a 1:1 mapping and that does not induce topological violations. One such measure is the distance from each boundary point to the boundary in a later frame. We mapped points in one frame (source points) to the boundary in a frame obtained 12 seconds later (target points), rather than to the one in the next frame, to reduce noise and to allow consistency across differing frame rates. Mapping each source boundary point to the closest target point in a later frame does not map boundary points into protrusions (*figure S5a*) and can lead to large gaps or clusters in the point sequence that is mapped to. Therefore, we smoothed over the list of target points. In other words, in each frame the mapping can be represented by a list in which the ith value is the index of the boundary point that boundary point i is mapped to. These lists were twice smoothed over using an averaging window (*figure S5b*). Notice that this method of smoothing does not induce topological violations. (The first smoothing had a window size of 19 boundary points, while the second had a size of 15 boundary points.) The magnitude of the local motion measure was then defined as the distance between a source point and the corresponding target boundary point in the smoothed list. If the target boundary point is outside of the current boundary, the local motion measure was defined to be positive; otherwise it was defined to be negative. As for the curvature measure, the local motion measure had a maximum value cut off for visualization. Also, boundary point labels were rotated, such that for each cell the greatest protrusive motion occurs at boundary point 300.

Analyzing Protrusive and Retractive Motion as Discrete Events

To analyze protrusive and retractive motion as discrete events, we calculated the location and the time of individual protrusions and retractions. First, we smoothed

the local motion measure with an averaging window of 3 frames and 3 boundary points. Next, in order to find protrusions, negative elements of the smoothed measure were set to zero. Elements with a value less than a noise threshold (1 pixel moved/12 seconds) were also set to zero. An individual protrusion was then defined as a peak in this processed local motion measure. Individual retractions were defined similarly.

Analyzing Protrusive and Retractive Motion as Continuous Boundary Movement

To analyze protrusive and retractive motion as continuous boundary movement, we found the mean protrusion and retraction locations in frames with boundary movement. Since the first boundary point is adjacent to the 400th boundary point, we used vector addition to calculate the mean values. For each boundary point, a vector was defined with direction $b*(2\pi/400)$, where b is the boundary point label. (Boundary points are labeled counter-clockwise from 1 to 400.) If the motion measure at a boundary point was positive, the magnitude of the associated vector was defined as the magnitude of the motion measure; otherwise, the magnitude was set to zero. We then defined the location of the mean protrusion as the direction of the sum of a frame's vectors multiplied by $400/2\pi$. If the motion measure at the mean location was below a noise threshold of one pixel, we did not define a mean location for that frame. The mean retraction location was found using an analogous procedure. *Figure 6c* shows extracted mean protrusion and retraction locations. Only mean locations that are defined in consecutive frames and that are less than 200 boundary points apart are shown.

We calculated the mean squared displacement (MSD) of the mean protrusion along the boundary (*figure 6d).* In the process of calculating the MSD, we found the distributions of the magnitude of mean protrusion displacement over various time intervals, which are shown in *figure S3*. Notice that from 4 seconds to 20 seconds the distributions flatten quickly, but from 20 seconds to 160 seconds, they flatten much more slowly.

Curvature Waves and the Cellular Footprint

To calculate the distance from the cell boundary to the cell footprint, we first extracted the outlines of the entire cell and of its footprint separately. In frames in which the footprint was composed of multiple disconnected regions, the snake was initialized surrounding all the regions, but upon iteration was allowed to pinch closed between the regions. The resultant snake then appears as outlined regions connected by thin lines. Points in these thin lines were excluded from further analysis by removing points that were extremely close to points with non-adjacent labels. A processed footprint boundary and a cell boundary are both shown overlaid on an IRM image sequence in *Movie 8.*

The distance from the cell boundary to the cell footprint was calculated for each point in the cell boundary. If a cell boundary point was inside the footprint boundary, the distance was defined as zero, otherwise, the distance was defined as the distance from that boundary point to the closest boundary point in the footprint

boundary. *Figure S4* shows the distance from the cell boundary to the cell footprint as a kymograph, while *figures 5a* and *c* show the cell boundary curvature and cell boundary motion kymographs for the same cell. Curvature waves, shown as white dashed lines, were drawn on the curvature kymograph and transferred to the distance kymograph. Notice that curvature waves tend to be initiated far from a region of surface contact, but, when they reach the side of the cell, are unusually close to a region of surface contact.

References

1. Baldacchini T, LaFratta CN, Farrer RA, Teich MC, Saleh BEA, et al. (2004) Acrylic-based resin with favorable properties for three-dimensional two-photon polymerization. Journal of Applied Physics 95: 6072-6076.
2. LaFratta CN, Baldacchini T, Farrer RA, Fourkas JT, Teich MC, et al. (2004) Replication of two-photon-polymerized structures with extremely high aspect ratios and large overhangs. Journal of Physical Chemistry B 108: 11256-11258.
3. Li L, Driscoll M, Kumi G, Hernandez R, Gaskell KJ, et al. (2008) Binary and gray-scale patterning of chemical functionality on polymer films. Journal of the American Chemical Society 130: 13512-13513.
4. Machacek M, Danuser G (2006) Morphodynamic Profiling of Protrusion Phenotypes. Biophysical Journal 90: 1439-1452.
